# Supplementary figures and images for: Transcriptome Analysis Reveals Differentially Expressed Genes Involved in Aluminum, Copper and Cadmium Accumulation in Tea ‘Qianmei 419’ and ‘Qianfu 4’
Source: Plants (Basel). 2023 Jul 7;12(13):2580. doi: 10.3390/plants12132580 (PMC10346602; doi:10.3390/plants12132580)

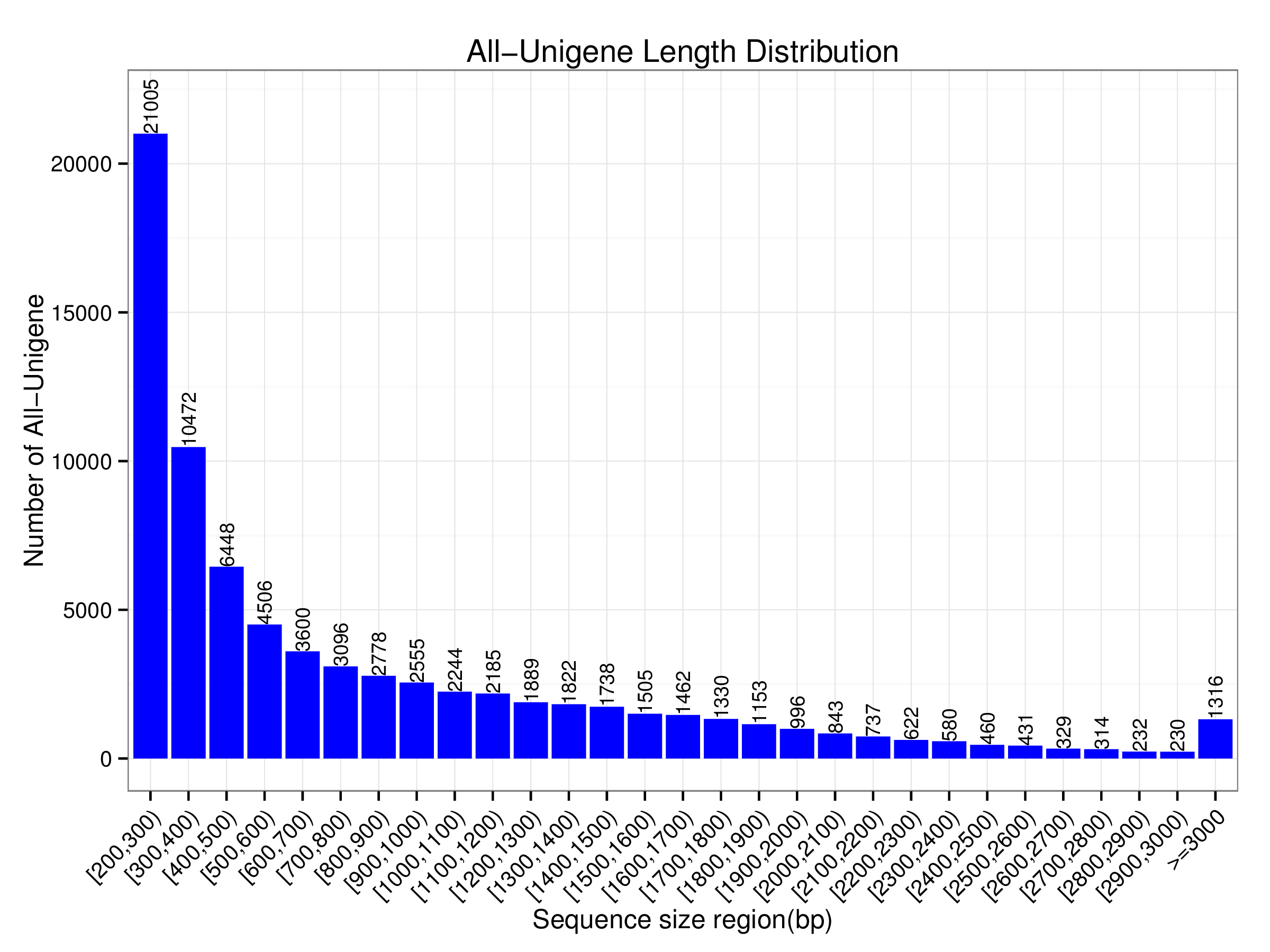

Supplement: Supplementary file 1 [file plants-12-02580-s001.zip › FIG S1 length of reads.png]

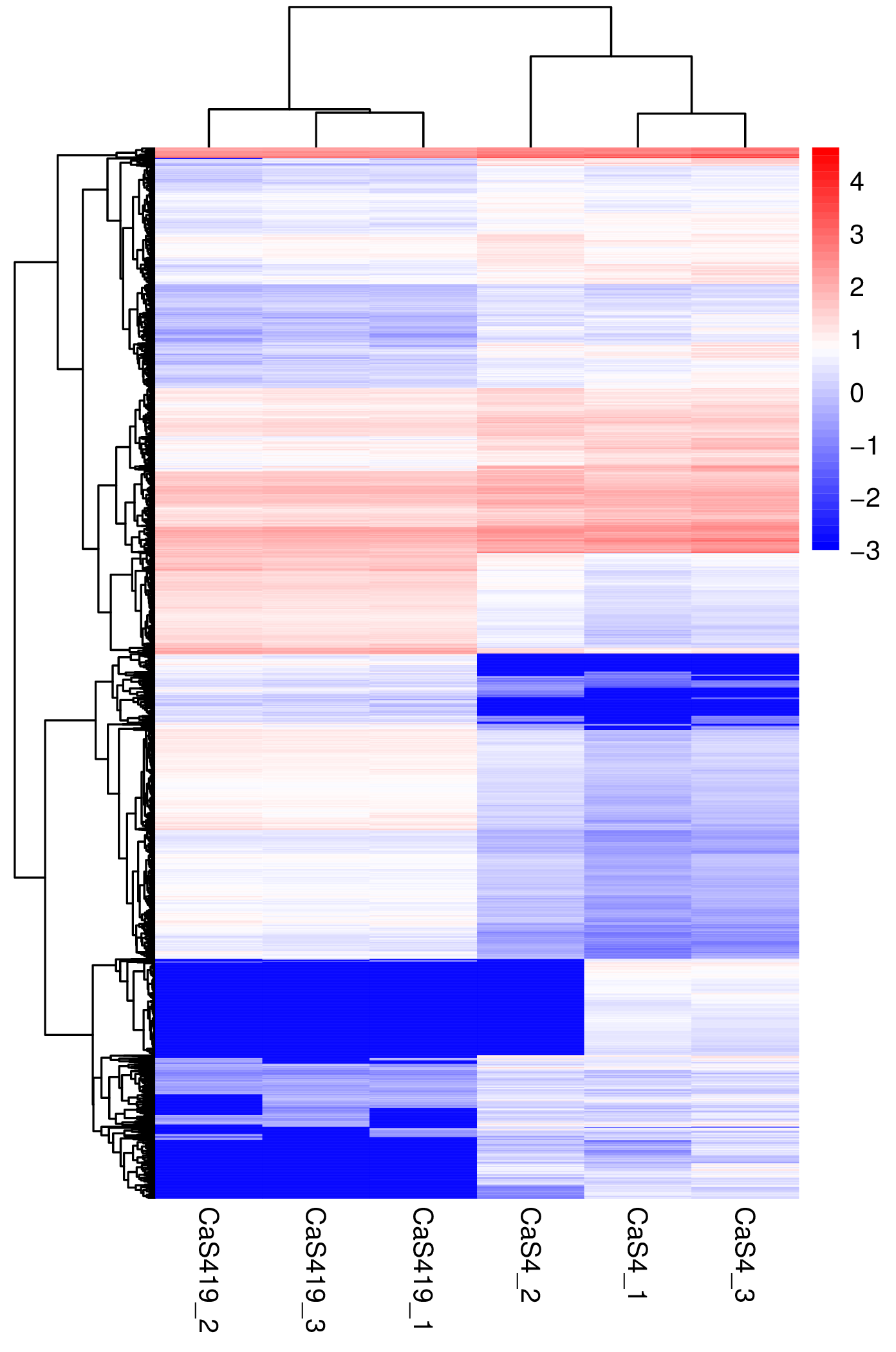

Supplement: Supplementary file 1 [file plants-12-02580-s001.zip › FIG S2 correlation between samples.png]
